# Challenges and Approaches to Establishing Multi-Pathogen Serosurveillance: Findings from the 2023 Serosurveillance Summit

**DOI:** 10.4269/ajtmh.24-0296

**Published:** 2024-09-03

**Authors:** Andrea C. Carcelen, Alex C. Kong, Saki Takahashi, Sonia Hegde, Thomas Jaenisch, May Chu, Rosemary Rochford, Natalya Kostandova, Emily S. Gurley, Amy Wesolowski, Andrew S. Azman, Fiona R. M. van der Klis, Gerco den Hartog, Christopher Drakeley, Christopher D. Heaney, Amy K. Winter, Henrik Salje, Isabel Rodriguez-Barraquer, Daniel T. Leung, Sammy M. Njenga, Eunice Wangeci Kagucia, Kondwani C. Jambo, Nicole Wolter, Richelle C. Charles, Martha-Idalí Saboyá-Díaz, Diana L. Martin, William J. Moss

**Affiliations:** ^1^ Department of International Health, Johns Hopkins Bloomberg School of Public Health, Baltimore, Maryland;; ^2^ Department of Epidemiology, Johns Hopkins Bloomberg School of Public Health, Baltimore, Maryland;; ^3^ Colorado School of Public Health, Aurora, Colorado;; ^4^ Geneva University Hospitals, Geneva, Switzerland;; ^5^ Center for Infectious Disease Control National Institute for Public Health and the Environment (RIVM), Bilthoven, The Netherlands;; ^6^ Laboratory of Medical Immunology, Radboud UMC, Nijmegen, The Netherlands;; ^7^ London School of Tropical Medicine and Health, London, United Kingdom;; ^8^ Environmental Health and Engineering Department, Johns Hopkins Bloomberg School of Public Health, Baltimore, Maryland;; ^9^ University of Georgia, Athens, Georgia;; ^10^ Department of Genetics, University of Cambridge, Cambridge, United Kingdom;; ^11^ Department of Medicine, University of California, San Francisco, California;; ^12^ Division of Infectious Diseases, University of Utah School of Medicine, Salt Lake City, Utah;; ^13^ Kenya Medical Research Institute (KEMRI), Nairobi, Kenya;; ^14^ KEMRI-Wellcome Trust Research Programme, Kilifi, Kenya;; ^15^ Malawi-Liverpool-Wellcome Programme (MLW), Blantyre, Malawi;; ^16^ Liverpool School of Tropical Medicine, Liverpool, United Kingdom;; ^17^ Centre for Respiratory Diseases and Meningitis, National Institute for Communicable Diseases of the National Health Laboratory Service, Johannesburg, South Africa;; ^18^ School of Pathology, Faculty of Health Sciences, University of the Witwatersrand, Johannesburg, South Africa;; ^19^ Massachusetts General Hospital, Harvard Medical School, Harvard T.H. Chan School of Public Health, Boston, Massachusetts;; ^20^ Department of Communicable Diseases Prevention, Control, and Elimination, Pan American Health Organization, Washington, District of Columbia;; ^21^ Division of Parasitic Diseases and Malaria, Centers for Disease Control and Prevention, Atlanta, Georgia

## Abstract

Multiplex-based serological surveillance is a valuable but underutilized tool to understand gaps in population-level exposure, susceptibility, and immunity to infectious diseases. Assays for which blood samples can be tested for antibodies against several pathogens simultaneously, such as multiplex bead immunoassays, can more efficiently integrate public health surveillance in low- and middle-income countries. On March 7–8, 2023 a group of experts representing research institutions, multilateral organizations, private industry, and country partners met to discuss experiences, identify challenges and solutions, and create a community of practice for integrated, multi-pathogen serosurveillance using multiplex bead assay technologies. Participants were divided into six working groups: 1) supply chain; 2) laboratory assays; 3) seroepidemiology; 4) data analytics; 5) sustainable implementation; and 6) use case scenarios. These working groups discussed experiences, challenges, solutions, and research needs to facilitate integrated, multi-pathogen serosurveillance for public health. Several solutions were proposed to address challenges that cut across working groups.

## INTRODUCTION

The WHO recently introduced collaborative surveillance as one of five interconnected components of health emergency preparedness, response, and resilience.^1^^,^^2^ A core objective of collaborative surveillance is to break down siloed disease surveillance systems and replace them with a collaborative and integrated system across diseases, public and private sectors, and administrative levels.

Serological surveillance, or serosurveillance, complements traditional public health surveillance for infectious diseases through the collection and analysis of specimens (e.g., serum, blood, or oral fluid) to measure antibodies to pathogens and estimate population-level exposure, susceptibility, and immunity to infectious diseases.^3^ This information can guide public health policies and programs for the control and elimination of several communicable diseases, including vaccine-preventable diseases (VPDs), neglected tropical diseases (NTDs), and emerging infectious diseases (EIDs). Although serosurveillance has been used for decades, the COVID-19 pandemic amplified interest in serology.^4^

The development of technologies such as multiplex bead immunoassays (MBIAs), which allow for the simultaneous detection of antibodies to more than one pathogen in a single assay, rapidly advanced the ability to efficiently conduct integrated, multi-pathogen serosurveillance.^3^^,^^5^ These technologies enable health systems to monitor exposure, susceptibility, and immunity to multiple pathogens with limited additional resources compared with using single-pathogen assays.^6^

Multiplex bead immunoassays have been developed for detecting antibodies against a range of pathogens including VPDs^7^^–^^9^; respiratory pathogens^10^; NTDs^11^^–^^15^; malaria^16^^,^^17^; sexually transmitted infections^18^; EIDs^19^; arboviruses^20^; and SARS-CoV-2.^21^ Integrating serosurveillance across multiple pathogens could efficiently leverage financial resources and personnel as well as metadata obtained from questionnaires. Integration with other surveillance programs (e.g., diagnostic, syndromic, and wastewater surveillance)^3^ could further improve the efficiency of surveillance systems to control the spread of disease.

Despite the MBIA being a powerful tool for public health surveillance, several barriers have prevented the widespread adoption of these technologies, particularly in low- and middle-income countries (LMICs), where they might be especially useful.^22^ Through partnerships with academic and government research institutions, multi-pathogen serosurveillance has been conducted in sub-Saharan Africa,^23^^,^^24^ Asia,^3^^,^^25^^,^^26^ and the Americas.^22^^,^^27^^,^^28^ The CDC and the Pan American Health Organization developed guidance for program managers to design and conduct integrated multi-pathogen serosurveillance.^28^

To discuss the opportunities and complexities with establishing integrated, multi-pathogen serosurveillance, experts from the Collaboration on Integrated Biomarkers Surveillance met in 2018 to catalog pathogens for inclusion in multiplex serological assays, lay out objectives for an integrated platform, identify potential use cases, and discuss advocacy.^29^ Building on this work, in March 2023, the International Vaccine Access Center at the Johns Hopkins Bloomberg School of Public Health, with support from the Bill and Melinda Gates Foundation and collaborators at the Center for Global Health at the University of Colorado Anschutz Medical Campus, convened a serosurveillance summit that further explored topics related to the use of MBIAs for multi-pathogen serosurveillance.

## SEROSURVEILLANCE SUMMIT MEETING

Experts across a range of institutions and fields participated, including researchers, multilateral organizations, country partners, private sector companies, and supply chain organizations. Participants were divided into two of six working groups: 1) supply chain, 2) laboratory assays, 3) seroepidemiology, 4) data analytics, 5) sustainable implementation, and 6) use case scenarios. The objectives of the workshop were to share experiences establishing integrated, multi-pathogen serosurveillance with a focus on MBIAs; identify key challenges, potential solutions, and research needs for integrated serosurveillance using MBIAs; and establish a community of practice of technical experts.

### Experiences and challenges.

**Figure 1.**
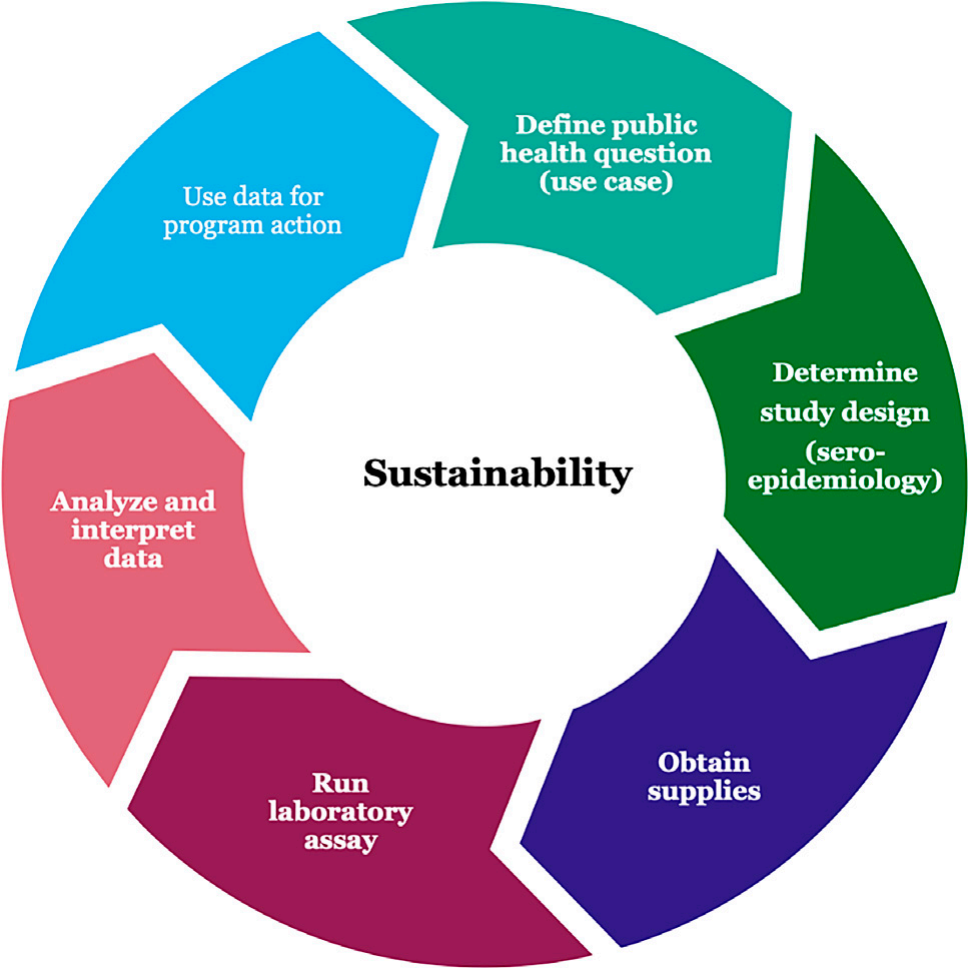
A framework depicting key steps for establishing a sustainable integrated serosurveillance system. The steps correspond with the six working groups, except for using data for program action, which is part of the sustainable implementation working group.

Participants shared their experiences with multi-pathogen serosurveillance (Annex 1). Based on these discussions, key issues that countries using multiplex serosurveillance have encountered were outlined using the steps for establishing a sustainable integrated serosurveillance system (Figure 1).

### Public health questions and use cases.

Because multi-pathogen surveillance involves multiple programs and partners, it can be challenging to generate buy-in from all stakeholders and identify potential programmatic impact early in the planning process. As more pathogens are included, additional groups working on different diseases will need to be engaged. Programmatic or research questions were framed as “use cases” for which serosurveillance is most likely to add value to existing surveillance systems. Table 1 presents five of the most common use cases identified and linked target pathogen(s) of interest. Several groups of pathogens were considered most relevant, including VPDs, emerging pathogens, NTDs, and other pathogens associated with high disease burden.

**Table 1:** Use cases for multiplex serosurveillance

Use Cases and a Representative Example	Example Pathogens
Estimating the burden and distribution of infections to complement or fill gaps in existing surveillance systems^30^	NTDs *Trypanosoma cruzi* (Chagas disease), Chikungunya Virus, *Taenia solium* (cysticercosis), *Strongyloides* spp., *Treponema pallidum* subspecies *pertenue* (yaws) Enteric pathogens *Campylobacter* spp., *Vibrio cholerae*, *Cryptosporidium*, *Giardia* Malaria *Plasmodium* Species HIV Respiratory Viruses Respiratory Syncytial Virus, Influenza Virus
Identifying emerging and reemerging infections^31^	Filoviruses Ebolaviruses, Marburg Virus Other Viruses Lassa Virus, mpox Virus SARS-CoV-2, Zika Virus
Identifying vaccine program reach or gaps and geographic or demographic gaps^32^	Childhood Diseases Measles Virus, Polioviruses, Rubella Virus, Diphtheria, Tetanus, Pertussis Other Viruses SARS-CoV-2, Yellow Fever Virus
Assessing changes in pathogen exposure due to behavioral, environmental, or (non-) pharmaceutical interventions or environmental changes^33^	NTDs Chikungunya Virus, Dengue Virus, Lymphatic Filariasis Bacteria *Streptococcus pneumoniae* spp., *Salmonella* serotype Typhi (Typhoid) Malaria *Plasmodium* Species
Monitoring peri- and post-elimination settings for diseases with elimination goals^34^	NTDs *Chlamydia trachomatis* (trachoma), *Dracunculus medinensis* (Guinea worm), *Leishmania* spp. (visceral leishmaniasis), Nematodes (lymphatic filariasis), *Onchocerca volvulus* (onchocerciasis), *Treponema pallidum* subspecies *pertenue* (yaws), *Trypanosoma brucei* (human African trypanosomiasis) Vaccine-Preventable Diseases Polioviruses Malaria *Plasmodium* spp. (subnational levels)

### Study design.

A key issue is identifying the appropriate target population for the question of interest. Because pathogens affect individuals across different demographic characteristics (e.g., age), selecting a target population to cover all pathogens of interest is challenging. Other challenges include defining population sampling strategies and sample sizes to answer multiple questions simultaneously and determining the optimal survey frequency to measure temporal trends across pathogens and address programmatic needs. One viable option includes nesting specimen collection within existing surveys that sample large populations across multiple characteristics (e.g., demographic and health surveys, multiple indicator cluster surveys). Less resource-intensive sampling strategies such as using residual blood specimens (e.g., from health facility laboratories or routine screening of pregnant women) can reduce costs while still capturing a range of populations and time points. In addition, specimen type (e.g., dried blood spots, venous blood, and oral fluid) must balance feasibility of collection and assay validity across all pathogens.

### Supplies.

One of the biggest challenges raised by multiple groups was related to the supply chain for assay reagents and equipment. Challenges included procuring, maintaining, and repairing platform technology, such as Luminex instruments. Procuring quality-assured beads and assay reagents in a space with few small-scale producers of conjugated beads is also an issue. Other challenges with supply chain sustainability involve understanding and addressing country-specific limitations to importation, maintaining the cold chain, and establishing procurement procedures and processes for reagents and supplies.

### Laboratory assays.

For multi-pathogen serosurveillance, several issues related to assay development, antigen discovery and validation, identification of proper controls, and assay performance must be addressed. Many research groups have developed standard operating procedures for equipment maintenance, assay techniques, assay development, quality control, and other key multiplex serology operations. However, resources such as positive and negative controls are not always available and require continuous support to laboratories for appropriate use. Furthermore, quality controls to validate assay runs and track assay performance over time are needed. Standardization holds the promise to make serosurveillance results more comparable between laboratories, but this is currently hampered by the limited availability of reference standards. Available reference reagents are typically calibrated for a specific pathogen, not for a broad range of them. Consequently, to report international units, standards need to be calibrated against each other, or an in-house standard must be built and calibrated against multiple standards.

Developing assays requires knowledge of the immunogenicity of antigens, kinetics of antibody responses, and relevance for assessing disease burden or protective immunity (i.e., correlates of immune protection). Because assay development can be technically challenging and requires thorough validation, the number of laboratories that currently undertake assay development is limited. High demand and inadequate capacity limit the availability of antigen-coupled beads, which are currently produced by a small number of research groups, although technology transfer initiatives are ongoing. For countries considering implementing multiplex serosurveillance but unable to develop assays, commercial options are also limited.

## STATISTICAL ANALYSES

There are currently no standardized approaches to cleaning raw laboratory data and establishing seropositivity thresholds, as this varies by antigen, the availability of controls, and population. This first stage of analysis includes performing quality control checks, evaluating serial dilution standard curves, and ensuring that steps to normalize data are appropriately applied. Translating cleaned data into useful epidemiological inference requires analytical approaches that model the normalized quantitative values or the establishment of reasonable cut-points for seropositivity that are relevant for the specific use case. In addition, the interpretation of pathogen-specific age patterns and geographic distributions of seroprevalence requires supplementing laboratory data with demographic characteristics and contextualized epidemiological understanding of the specific pathogens. The use of serological data for computing age seroprevalence curves and estimating epidemiological parameters, such as forces of infection, is established for well-characterized antigens. A key remaining challenge is selecting and implementing appropriate analytical approaches (and metadata) to answer questions of interest for less–well-characterized antigens as well as across multiple antigens or pathogens simultaneously. Furthermore, there is a need for data analytic pipelines to facilitate the interpretation of data by balancing detail and complexity and producing user-friendly data visualizations such as spatial maps.

### Data for program action.

Given the complexities of analyzing multiple pathogens simultaneously, it can be challenging to generate interpretable and easily visualized results for target audiences such as policymakers and program managers. The time from serosurvey data collection to dissemination of results is often long (months), which makes it challenging to use results for decision-making. Serosurvey data should be triangulated with other data sources for interpretation of the epidemiological findings for each disease. When serosurveillance for several pathogens is simultaneously analyzed and presented, it can be challenging to contextualize all findings in a succinct, clear manner.

### Cross-cutting challenges.

Training needs include supply chain logistics and procurement so laboratories can place their supply orders and anticipate shortages. Similarly, training on instrument use and routine maintenance are needed to sustain high instrument performance and minimize downtime and costly repairs. There are also challenges with technology transfer, including training on the MBIA, bead coupling and validation, and quality control procedures. In addition, there is a strong need for training in data analytics, as multi-pathogen serosurveillance data are complex and most useful when combined with complementary data such as vaccination coverage or case-based disease surveillance data.

Table 2 summarizes challenges identified in each working group. Additional details are available in the full meeting report.^35^

**Table 2:** Key challenges identified by the working groups

Working Group	Key Challenges
Supply chain	Procuring and maintaining appropriate platform technology, producing and procuring quality-assured beads and assays, commercializing kits, maintaining the cold chain, understanding and addressing country-specific limitations to importation, and limited human and technological capacity to anticipate and avoid supply chain issues
Seroepidemiology	Selecting sample populations and sample sizes, establishing the frequency of sampling, identifying and validating less resource-intensive sampling strategies, defining sampling approaches that answer multiple questions, determining core individual- and household-level data to collect, and linking serosurvey antigens to study design for programmatic impact
Laboratory assays	Supporting technology transfer and training, sharing best practices and protocols, standardizing antigen use across countries, defining quality control standards, and developing reference reagents
Data analytics	Standardizing and cleaning raw laboratory data, translating cleaned data into useful epidemiological inference by selecting appropriate analytical approaches to answer questions of interest, and developing user-friendly analytical and visualization pipelines
Sustainable implementation	Demonstrating added value for initial engagement, generating buy-in across national health systems, ensuring adequate laboratory capacity and procurement, and interpreting data and integrating results for decision-making

### Proposed solutions across working groups.

#### Creation of an electronic platform to share resources and expertise.

There is a strong need for information-sharing platforms. New tools and resources needed to support multiplex serosurveillance include a planning tool that would allow users to prepare for resources needed and to estimate cost; a supply chain “playbook” that details cold chain and labeling requirements and substitutable reagents; protocols that enable adaptive sampling strategies; and ethical considerations for additional testing in serosurveillance studies. Furthermore, case studies demonstrating how countries have used serosurveillance to guide public health actions would help underscore the value of serosurveillance. In addition, the platform could aid in standardizing preprocessing pipelines between studies and harmonizing data.

A unified platform could host these tools and other resources such as a central repository for antigens and standards, protocols, data packages and scripts, sample size calculation tools, best practices, training resources, and plain-language policy briefs and technical documents. Critically, this platform should also allow users to communicate with one another to troubleshoot problems and share experiences. Creating a single platform to address these needs could help develop and sustain a culture of collaboration while facilitating harmonization efforts where practical.

#### Building local capacity and training.

Expanding the capacity for MBIAs in low-resourced settings will help generate data where they are most needed. Enhanced capacity could also create a more favorable environment for commercialization, enable greater collaboration and country ownership, promote harmonization, and address key bottlenecks. Several areas were identified as priorities for capacity building, including the development of regional hubs and use of multiple training approaches such as on-site training, online training, and train-the-trainer initiatives. These approaches could enable users to perform MBIAs, service and troubleshoot bead-based multiplex platforms, and produce or procure antigen-coupled beads. Although discussion of capacity building focused on LMICs, many areas were relevant for users in all countries.

#### Developing quality control and standardized approaches.

Exploring ways to standardize approaches would allow for comparison of results across countries. However, harmonization can be challenging. Targets for standardization include standardizing approaches to conducting serosurveys; creating or procuring quality assay materials; and best practices for cleaning, analyzing, and presenting data. Developing and validating positive and negative reference controls by antigen (e.g., through the United Kingdom’s National Institute for Biological Standards and Control or using validated recombinant antibodies) would lead to results that are more interpretable across assays, populations, and time points. Developing a common panel with the most frequently used antigens across regions could also facilitate cross-country comparisons, though customization would still be needed to address country-specific priorities.

#### Establishing a laboratory network and building partnerships.

A laboratory support network would facilitate knowledge sharing and troubleshooting at country, regional, and global levels, helping to connect laboratory groups. Partnering with private companies would support commercialization of panels and sharing of know-how regarding supply chain constraints. Partnering with supply chain experts would enable procurement and packaging of common reagents and materials to streamline ordering processes and avoid delays caused by stockouts. Regional networks could also allow groups to share limited resources—including access to instruments and materials like antigen-coupled beads—and to pool demand for these resources. Regional hubs could be characterized by function (e.g., coupling antigens to beads and providing quality control) to help meet the needs of different groups, further building a collaborative network.

#### Generating political buy-in for multiplex serosurveillance.

Participants viewed the establishment of buy-in from governments, funders, and regulatory agencies as essential for the introduction and scale-up of multi-pathogen serosurveillance. Approaches to achieving support and fostering greater participation from these entities include exploring standardized approval processes for the importation of products necessary for multi-pathogen serosurveillance, developing a taxonomy of pathogen-specific antigens paired to scientific and policy-relevant use cases, involving governmental agencies in training initiatives, and developing analytical and visualization pipelines to aid understanding. Garnering high-level regional and international support to develop guidance and recommendations for the implementation and use of integrated serosurveillance was considered a priority. Organizations such as the *Pan American Health Organization* (PAHO) and the U.S. CDC have developed documents that were discussed as starting points.^28^ This goal could be supported through conversations with decision-makers to demonstrate how integrated, multi-pathogen serosurveillance can complement existing disease surveillance systems and by providing successful case studies. Generating community buy-in through communication of the benefits and limitations of serosurveillance is also critical, as exploring the value of integrated serosurveillance hinges on their participation.

## DISCUSSION

Serosurveillance provides valuable information to guide public health programs, especially when triangulated with data from other surveillance systems. In isolation, serosurveillance systems are costly to establish and sustain.^28^^,^^36^ Serosurveillance data are underutilized because of the heterogeneity of assays and the delay in disseminating results to health authorities for meaningful program impact.^37^^,^^38^ Ideally, integration of serosurveillance with routine public health activities can reduce costs and make it more sustainable, but that requires sufficient buy-in and funding.^6^^,^^14^^,^^23^^,^^39^ The lessons learned from experiences establishing serosurveillance across multiple countries should be shared to promote further investment in this technology.

For serosurveillance to have programmatic impact, data must be available in a timely fashion. Several bottlenecks cause delays: planning epidemiologically relevant serosurveys; procuring materials and equipment; and cleaning, analyzing, and interpreting data.^29^ Some approaches, such as developing standard operating procedures, addressing supply chain issues, optimizing data analysis pipelines, training local health researchers, and sharing preliminary results with decision-makers can shorten the time for data to be used for action.^22^ Timely serosurveillance data provide insights into disease transmission patterns and population vulnerability to outbreaks to guide control and elimination strategies.

Financial, technical, and political support is also needed to overcome these bottlenecks. For example, the development of a commercial panel for frequently tested antibodies could address supply chain constraints, but commercialization restricts flexibility to modify the pathogens that can be tested. For commercialization of panels, there will need to be sufficient demand. Without adequate resources, serosurveillance efforts may only be pilots or ad hoc endeavors. Investment in the development of country-led, multi-pathogen serosurveillance systems such as PAHO’s^28^ can expand the number of countries conducting multi-pathogen serosurveillance.

In addition to the use cases presented, there are additional questions of public health importance that could be explored (e.g., optimizing vaccination schedules). Recently, the most common use case was measuring the spread of SARS-CoV-2; seroprevalence studies were conducted in 149 countries.^4^ This allowed tracking the spread of the virus, identifying transmission dynamics, monitoring population immunity, and evaluating vaccine program performance.^40^^,^^41^ Leveraging the capacity building, networking, platforms, and expertise developed during the COVID-19 pandemic could better prepare us for the next emerging pathogen and support surveillance systems for diseases that are underfunded.

The global response to the COVID-19 pandemic also demonstrated the power of coordination across institutions. Monitoring seroprevalence and population immunity in different settings harnessed learnings across the globe. Although harmonized approaches were feasible for SARS-CoV-2 and allowed for cross-country comparisons, many pathogens need additional research to allow for such comparisons. Vaccine-preventable diseases such as measles and rubella already have standardized international controls, agreed upon correlates of protection, existing laboratory networks, and clear programmatic actions that can be informed by serological data.^42^ As multiplex panels are developed for different pathogens, similar standardization could enable results to be more readily compared across settings. Although VPDs are an area where standardization is within reach, achieving this aim across a diverse array of pathogens—especially considering the unique epidemiological profiles and priorities of different countries—will require more developed serosurveillance systems and international coordination.

Although multi-pathogen serosurveillance has traditionally been used in high-income countries (HICs),^43^ it has also been used in LMICs, often with a high degree of technical support from organizations based in HICs. Some studies include samples from LMICs that were tested entirely in an HIC,^44^ in both LMICs and HICs,^21^^,^^45^ and entirely in an LMIC.^23^^,^^46^^–^^49^ To ensure the promotion of country ownership, initiatives are needed to build local capacity to couple beads, perform MBIAs, and analyze data that are coordinated with national governments and aligned with their priorities. More recently, efforts to transfer technology and build capacity in countries in the Americas^22^^,^^28^ and Africa^23^ have paved the way for future endeavors to scale up multiplex serosurveillance. To move toward routine serosurveillance globally, additional funding is needed to fill research gaps and advance implementation in additional settings, including bolstering capacity in laboratories that do not yet have the technologies used in multi-pathogen serosurveillance.

Building on the momentum from previous efforts, the 2023 Serosurveillance Summit provides further impetus to advance collaboration among countries to conduct multi-pathogen serosurveillance. Participants will continue serving on working groups to put into practice the proposed solutions outlined above. This community of practice brings together a network of scientists and practitioners to facilitate knowledge sharing and develop a platform for multi-pathogen, multi-country serosurveillance. These established networks and relationships could facilitate rapid response efforts for future emerging pathogens. As the world moves to reclaim the progress against infectious diseases that was disrupted by the COVID-19 pandemic—and to enhance preparedness to prevent or mitigate the next pandemic—the appetite for establishing multi-pathogen serosurveillance systems has never been greater.

## Supplemental Materials

10.4269/ajtmh.24-0296 Supplemental Materials
