# Supplementary material for: Challenges and Approaches to Establishing Multi-Pathogen Serosurveillance: Findings from the 2023 Serosurveillance Summit
Source: Am J Trop Med Hyg. 2024 Sep 3;111(5):1145–52. doi: 10.4269/ajtmh.24-0296 (PMC11542533; doi:10.4269/ajtmh.24-0296)
Supplement: Supplemental Materials [file tpmd240296.SD1.pdf]

Annex 1. Select experiences shared by participants during the Serosurveillance Summit

Working Group	Selected Relevant Experiences
Use Case Scenarios	One example was an integrated serological survey which included several diseases with different objectives. The survey included ten different pathogens (lymphatic filariasis [a target for elimination], malaria, diseases for which they did not have much data available [e.g., toxoplasmosis and others], and four VPDs) in hard-to-reach populations. It was important to understand the gaps and what interventions need to be designed.<br><br>There was an example of a study in a city in a coastal region, known to have dengue outbreaks from time to time. The counties are decentralized, so each county is responsible for their health. The county did not have the capacity to test for dengue in facilities except at the hospital, that had run out of tests. They were using clinical diagnosis aside from the rapid test if available (if the case presented was not malaria, then it was classified as dengue). When they performed a dengue antibody test, they found 80% seropositivity.
Supply Chain	One participant shared an experience of not receiving supplies that were ordered for 4 months despite paying on time. They eventually discovered that the supplier had not yet been able to procure a few items from the order and did not want to ship anything until the order was complete, despite the materials being easily substitutable items like bottles and labels.<br><br>Several participants noted difficulties with importing certain reagents ranging from temperature-sensitive materials sitting in customs for extended periods of time and rendering them inactive, to deciding not to include certain antigens like tetanus toxoid in their work because stringent importation standards would not allow its entry as a product that could be used in biological warfare. Some participants noted that in their countries, all reagents and lab consumables are imported because nothing is manufactured locally. They described additional experiences related to importation including having to pay unexpectedly exorbitant prices for reagents due to dramatic fluctuations in currency exchange rates while international payments were waiting to clear.
Seroepidemiology	Participants described their different experiences and approaches to sampling populations for serosurveys. This included school-based sampling, residual specimens from healthcare facilities, biorepository specimens, and specimens from members of a population randomly selected to receive a collection kit by mail that they then sent back.

Laboratory Assays	One participant described a regional approach to developing laboratory assay capacity by sending trainers to national laboratories, grouping countries by region, and sharing both standard operating procedures and samples among the regions. In this way, resources could go further.<br><br>Another participant described efforts to ensure adequate quality control by sharing their antigen-bead coupling procedure, controls, and standards with other countries and going to these countries to train personnel. They further support this quality by checking for inter-batch variability by validating every batch of antigen-coupled beads against the same samples.
Data Analytics	Several participants indicated that they had developed and used their own RShiny apps for data analysis, including one which allowed users to import the Luminex results of an MBIA with controls to provide quality control, data wrangling, and estimating seropositivity based on a binarized outcome. However, these apps varied between groups.<br><br>Participants noted that establishing cut-offs for some pathogens can be particularly difficult, especially when previous work on these pathogens is limited. For SARS-CoV-2, multiple challenges were discussed, including the use of specimens from hospitalized COVID-19 patients to establish cut-points leading to overly high thresholds. Additionally, these values differed between commercial assays.
Sustainable implementation	Community members have not always understood the purpose or value of participating in serosurveys. In one situation, the local teams were not adequately trained to explain this to the community. In another, individual community members declined to provide samples because they believed that they were in good health, and participation did not confer a personal benefit. Some participants shared experiences where these benefits were provided in the form of tests like blood glucose testing for diabetes or other measurements to diagnose non-communicable diseases, which resulted in strong participation rates.<br><br>A noted challenge experienced building local capacity to run MBIAs was that researchers might attend trainings in other countries only to return to a major backlog of enzyme-linked immunosorbent assays (ELISAs) testing for surveillance. This backlog required dedicated, full-time work for several months, after which some of the training was forgotten.<br><br>One participant noted that they had previously received excellent instrument maintenance service from a technician who would travel to their lab from another country. Finding reliable local service has since dwindled, and groups have struggled to find well-trained service providers.

JOHNS HOPKINS BLOOMBERG SCHOOL of PUBLIC HEALTH

INTERNATIONAL VACCINE ACCESS CENTER

Serosurveillance Summit 2023

Bloomberg School of Public Health, Baltimore, Maryland
March 7 - 8, 2023

Working Groups

Overview

Each meeting day is broken into two sessions, during which discussion will be conducted regarding the thematic areas. The overall purpose of these working groups are:

1. To identify the general challenges in multiplex integrated serological surveillance related to each topic area with a focus on existing technologies, particularly multiplex bead arrays.
2. To establish a community of practice to tackle issues for multiplex serology going forward.

Everyone has been placed into two working groups that focus on a thematic area, with the consideration of your preferences and experiences. The leaders of your working groups should reach out to you before the summit with a list of key challenges identified related to the group's thematic area. Please review these challenges for feedback and think about cross-cutting, as opposed to pathogen-specific, solutions & approaches to discuss during the summit.

Group Descriptions

Supply Chain

This group will focus on supply chain issues including multiplex bead array assay availability, reagent manufacturing, equipment, and opportunities for technology transfer.

- Availability of antigen coupled beads
- Barriers to commercial manufacturing of assay reagents
- Assay and antigen validation
- Opportunities for technology transfer
- Affordability and economic considerations

Laboratory Assays

This group will address thresholds for seropositivity, standardization, control panels, methods and tools for processing, considerations for improved assays for certain antigens (cross reactivity, antibody kinetics, etc).

- Ensuring quality standards
- Establishing thresholds for seropositivity
- Assay validation across antigens

- Standardization across countries to ensure comparability
- Access to positive and negative control panels
- Improved assays for selected antigens (addressing cross reactivity, antibody kinetics, etc.)

Seroepidemiology

This group will look at epidemiologic considerations such as study design, sampling strategies, handling different target populations, and ways to address biases.

- Survey design for multiplexed serosurveillance
- Specimen sampling strategies (residual, cross-sectional, longitudinal)
- Target populations by demographic characteristics across antigens
- Sample size issues for each antigen
- Addressing potential biases

Data Analysis

This group will assess analytical approaches to seroprevalence data, combining modeling with seroprevalence data, approaches to data triangulation, and tools to support in-country analysis.

- Standardized analytical approaches to analyzing seroprevalence data across antigens
- Combining modeling with seroprevalence data
- Approaches to data triangulation
- Data sharing and platforms for collaborations
- Tools for supporting in-country analysis

Use Case Scenarios

This group will look at use cases for serosurveillance across antigens in terms of how serology informs programmatic decision making, building off epidemiological scenarios for integrated serosurveillance.

- Use cases across antigens for programmatic decision making
- Pan-national issues
- Pathogen priority list
- PAHO’s epidemiological scenarios for integrated serosurveillance

Sustainable Implementation

This group is focused on country issues related to implementation, policy implications and sustainability of serosurveillance systems. This includes dissemination and translation of results to policymakers, and challenges in establishing integrated serosurveillance systems.

- Dissemination and translation of serosurvey results to policy makers
- Training and resource needs
- Challenges and opportunities in establishing sustainable, integrated serosurveillance systems

- PAHO experience: political engagement, technical involvement, innovative planning
- Regional laboratory networks
- South-south collaboration

Group Members

	Sustainable Implementation	Lab Assay	Seroepidemiology
**Leads**	Martha Idali Saboya-Diaz<br>Fiona van der Klis<br>Sammy Njenga<br>Amy Wesolowski	Christopher Heaney<br>Diana Martin<br>Gerco den Hartog<br>Rosemary Rochford	Eunice Wangeci Kagucia<br>Andrew Azman*<br>Sonia Hegde<br>Nicole Walter
**Notetaker**	Julia Poje	Lindsay Avolio	Shahjahan Ali
**Members**	Alison Jones<br>Amy Winter<br>Bryan Grenfell*<br>Emily Gurley<br>Hellen Gelband<br>Gretchen M Cooley*<br>Ibrahim Bob Swaray<br>Jonathan Jasson Mandolo<br>Juliet Bryant*<br>Leanne Robinson*<br>Upendo Lisa Mseka<br>Mairead Whelan<br>Manoj Vasant Murhekar<br>May Chu<br>Megan O'Driscoll<br>Melissa Richard-Greenblatt*<br>Yannik Roell	Bharat Parekh*<br>Catriona Patterson<br>Daniel T. Leung<br>Fiona Angrisano*<br>James Nyagwange*<br>Jill Ray<br>Kevin Tetteh<br>Kokou Nouwame<br>Kondwani Jambo<br>Makhtar Niang<br>Mattie Cassaday<br>Nora Pisanic<br>Ramee Saleh<br>Rhea Longley*<br>Richelle Charles<br>Ross Kedl<br>Samantha Dolan<br>Sophie Berube<br>Taufiqur Rahman<br>Wilhelmina Strasheim	Arthur Menezes<br>Ben Arnold<br>Cheryl Cohen*<br>Christopher Drakeley<br>Derek Cummings<br>Eric Rogier<br>Godfrey Bigogo<br>Henrik Salje<br>Isaac Ssewanyan<br>Isabel Rodriguez<br>Ivo Mueller*<br>Jordan Tappero<br>Kirsten E. Wiens*<br>Kristen Aiemjoy<br>Kristin Savage<br>Shazia Ruybal*<br>Thebora Sultane<br>Thomas Jaenisch

	Supply Chain	Data Analysis	Use Cases
**Leads**	May Chu<br>Daniel T Leung<br>Richelle Charles	Saki Takahashi<br>Amy Winter<br>Henrik Salje<br>Isabel Rodriguez	Thomas Jaenisch<br>Kondwani Jambo<br>Emily Gurley<br>Christopher Drakeley
**Notetaker**	Alex Kong	Mattie Cassaday	Natalya Kostandova
**Members**	Alexandra Morel<br>Aloysius Bingi<br>Catriona Patterson<br>Bharat Parekh*<br>Blake Puneky<br>Eddie Alberado<br>Fiona Angrisano*<br>Fiona van der Klis<br>Hellen Gelband<br>Jill Ray<br>Jonathan Jasson Mandolo<br>Julia Poje<br>Kevin Tetteh<br>Kokou Nouwame<br>Ramee Saleh<br>Rhea Longley*<br>Rosemary Rochford<br>Samantha Dolan<br>Shahjahan Ali<br>Thebora Sultane<br>Wilhelmina Strasheim	Andrew Azman*<br>Ben Arnold<br>Bryan Grenfell*<br>Christopher Heaney<br>Derek Cummings<br>Gerco den Hertog<br>Godfrey Bigogo<br>Gretchen M Cooley*<br>Ibrahim Bob Swaray<br>Kirsten E. Wiens*<br>Kristin Savage<br>Manoj Vasant Murhekar<br>Megan O'Driscoll<br>Nicole Wolter<br>Ross Kedl<br>Sophie Berube<br>Taufiqur Rahman Bhuiyan	Alison Jones<br>Cheryl Cohen*<br>Diana Martin<br>Eric Rogier<br>Eunice Wangeci Kagucia<br>Isaac Ssewanyan<br>Jordan Tappero<br>Juliet Bryant*<br>Kristen Aiemjoy<br>Leanne Robinson*<br>Mairead Whelan<br>Makhtar Niang<br>Martha Idali Saboya-Diaz<br>Melissa Richard-Greenblatt*<br>Sammy Njenga<br>Shazia Ruybal*<br>Sonia Hegde<br>Upendo Lisa Mseka<br>Yannik Roell

*Attending virtually via Zoom

Agenda

Objectives

Discuss experience with establishing integrated multiplexed serosurveillance systems
Discuss challenges in establishing integrated multiplexed serosurveillance systems
Discuss opportunities to expand integrated multiplexed serosurveillance systems
Identify research needs for integrated multiplexed serosurveillance systems
Establish community of practice for integrated multiplexed serosurveillance systems

Day 1: Discussing Solutions & Approaches

Tuesday, March 7, 2023

8:30 AM – 9:00 AM

Check In
Bloomberg School of Public Health, East Monument Street entrance
Meet at the East Monument Street entrance to check-in, receive your badge, and locate the meeting room.

9:00 AM – 10:00 AM

Welcome Address
Feinstone Hall | Zoom Link – Meeting ID 91692618844 (Passcode 570946)
Organizers will give a welcome address, followed by introductions and setting the objectives of the workshop.

Dr. William Moss, Johns Hopkins University, *Welcome & Goals*
Dr. May Chu, University of Colorado, *Center for Global Health Consortium*
Dr. Eunice Kagucia, Kenya Medical Research Institute (KEMRI), *Country Perspective on Setting up Integrated Serosurveillance*
Dr. Marc Bulterys, Bill and Melinda Gates Foundation, *Vision for Integrated Serosurveillance*
Ambassador Dr. John Nkengasong, U.S. Department of State
Dr. Andrea Carcelén, John Hopkins University, *Meeting Overview & Objectives*

10:00 AM – 10:30 AM

Break & move to working group meeting rooms

10:30 AM – 12:30 PM

Morning Working Session - Part 1
Seroepidemiology - Room W2017 | Zoom Meeting 94531017769 (Passcode 171471)
Lab Assay - Room E9519 | Zoom Meeting 92491075304 (Passcode 113842)
Sustainable Implementation - Room W3031 | Zoom Meeting 94113538479 (Passcode 291028)
Working groups will meet to review the key challenges identified in their thematic areas by the co-leads and make any additions. Attendees will discuss how groups have addressed these challenges in the past and describe the context in which they were used. Attendees

will also brainstorm new potential alternative solutions that address these challenges. The rapporteur will take minutes of the discussion, particularly the lessons learned and solutions, and should keep a list of the resources named during this session. Discussion will be synthesized into approaches and solutions to challenges using a template.

**12:30 PM – 1:30 PM**	**Lunch Break**<br>Feinstone Hall
**1:30 PM – 3:30 PM**	**Afternoon Working Session - Part 1**<br>**Supply Chain** - Room E9519 | Zoom Meeting 97270138502 (Passcode 491863)<br>**Data Analysis** - Room W3031 | Zoom Meeting 97170883881 (Passcode 458858)<br>**Use Case Scenarios** - Room W2017 | Zoom Meeting 94107817980 (Passcode 026280)<br>During this session, individuals will join a different working group. Working groups will discuss the same as above.
**3:30 PM – 3:45 PM**	**Break & return to main room**
**3:45 PM – 5:00 PM**	**Working Group Reports**<br>Feinstone Hall | Zoom Link – Meeting ID 91692618844 (Passcode 570946)<br>Working groups will present on progress, challenges, overlap with other working groups, and key takeaways from the day (10 min each).
**5:30 PM**	**Social Event (optional)**<br>Ministry of Brewing<br>1900 East Lombard Street<br>Baltimore, MD 21231

Day 2: Developing a Plan
Wednesday, March 8, 2023

**8:30 AM – 9:00 AM**	**Check-In & Coffee**<br>Feinstone Hall<br>Arrive at Bloomberg School of Public Health for coffee and muffins. Badges provided on the first day should permit building entry for both days.
**9:00 AM – 10:00 AM**	**Welcome & Instruction**<br>Feinstone Hall | Zoom Link – Meeting ID 91692618844 (Passcode 570946)<br>After an overview of the previous day, key objectives for the second day will be outlined.
**10:00 AM – 10:15 AM**	**Group Photo**
**10:15-10:30**	**Break & move to working group rooms**
**10:30 AM – 12:30 PM**	**Morning Working Session - Part 2**<br>**Seroepidemiology** - Room W2017 | Zoom Meeting 94531017769 (Passcode 171471)<br>**Lab Assay** - Room E9519 | Zoom Meeting 92491075304 (Passcode 113842)<br>**Sustainable Implementation** - Room W3031 | Zoom Meeting 94113538479 (Passcode 291028)<br>Working groups will meet to review the challenges and potential solutions identified during day 1. The goal is to develop an agenda for next steps to move multiplex serosurveillance forward. They will also prepare the summaries of challenges, solutions, and gaps to report to the larger group in the wrap-up session. Specific objectives during this session will be to:<br>1. Review list of potential new solutions defined during first working group session and identify whether these are short-term or long-term solutions based on their feasibility<br>2. Evaluate the challenges and prioritize approaches currently being used and proposed solutions to determine the next steps to address these challenges. This could include scaling up approaches already being used, implementing a proposed solution, or defining additional needs.<br>*Conceptualize next steps as an advocacy pitch or setting a research agenda.*<br>3. Review list of approaches described during first working group session and describe their limitations. *(If time allows)*
**12:30 PM – 1:30 PM**	**Lunch Break**<br>Feinstone Hall
**1:30 PM – 3:30 PM**	**Afternoon Working Session - Part 2**<br>**Supply Chain** - Room E9519 | Zoom Meeting 97270138502 (Passcode 491863)<br>**Data Analysis** - Room W3031 | Zoom Meeting 97170883881 (Passcode 458858)<br>**Use Case Scenarios** - Room W2017 | Zoom Meeting 94107817980 (Passcode 26280)

During this session, individuals will join their second working group. Working groups will discuss the same as above.

**3:30 PM – 3:45 PM**	**Break & return to main room**
**3:45 PM – 4:45 PM**	**Working Group Reports**<br>Feinstone Hall | Zoom Link - Meeting ID 91692618844 (Passcode 570946)<br>Each working group will take 10 minutes to report out on their findings and solutions from the summit and identify key next steps.
**4:45 PM – 5:00 PM**	**Closing Remarks**<br>Feinstone Hall | Zoom Link - Meeting ID 91692618844 (Passcode 570946)
